# Interindividual Variability in Functional Connectivity as Long-Term Correlate of Temporal Discounting

**DOI:** 10.1371/journal.pone.0119710

**Published:** 2015-03-16

**Authors:** Cinzia Calluso, Annalisa Tosoni, Giovanni Pezzulo, Sara Spadone, Giorgia Committeri

**Affiliations:** 1 Department of Neuroscience, Imaging and Clinical Sciences, G. D’Annunzio University, Via dei Vestini 33, 66013, Chieti,Italy; 2 Institute for Advanced Biomedical Technologies, G. D’Annunzio Foundation, Via dei Vestini 33, 66013, Chieti, Italy; 3 Institute of Cognitive Sciences and Technologies, CNR, via S. Martino della Battaglia 44, 00185, Roma, Italy; Leibniz Institute for Neurobiology, GERMANY

## Abstract

During intertemporal choice (IT) future outcomes are usually devaluated as a function of the delay, a phenomenon known as temporal discounting (TD). Based on task-evoked activity, previous neuroimaging studies have described several networks associated with TD. However, given its relevance for several disorders, a critical challenge is to define a specific neural marker able to predict TD independently of task execution. To this aim, we used resting-state functional connectivity MRI (fcMRI) and measured TD during economic choices several months apart in 25 human subjects. We further explored the relationship between TD, impulsivity and decision uncertainty by collecting standard questionnaires on individual trait/state differences. Our findings indicate that fcMRI within and between critical nodes of task-evoked neural networks associated with TD correlates with discounting behavior measured a long time afterwards, independently of impulsivity. Importantly, the nodes form an intrinsic circuit that might support all the mechanisms underlying TD, from the representation of subjective value to choice selection through modulatory effects of cognitive control and episodic prospection.

## Introduction

Daily decisions often involve a tradeoff between the chance of earning a smaller but immediate reward or a larger but delayed reward. In such types of choices, in which gains are evaluated against time, future outcomes are typically devaluated as a function of the delay. This phenomenon, known as temporal discounting (TD), is well exemplified by the famous saying “a bird in the hand is worth two in the bush”. Temporal discounting represents a highly idiosyncratic function, i.e. the steepness of the discounting curve shows deep differences across subjects, and is also relatively stable over time [1]. Moreover, as recently reviewed by Odum [2], there are at least three lines of evidence supporting the idea that discounting behavior can be considered a personality trait: delay discounting, as other personality characteristics, can change with maturity as part of normal developmental and experiential adaptation; it is correlated across different decision domains; and it appears to be highly heritable.

Importantly, because of its pervasiveness in daily life decisions and its relevance for several disorders, such as pathological gambling [3,4], substance abuse [5–7] and attention-deficit hyperactivity [8,9], TD is among one of the most studied topic in behavioral neuroscience. In particular, based on the analysis of neural activity during intertemporal choice paradigms, in which individuals are required to choose between an immediate smaller reward and a larger delayed reward, at least two main neural accounts have been proposed. One account, which is directly inspired by the quasi-hyperbolic model of discounting, considers intertemporal choice as the result of the interaction between two competing systems, one specialized to value immediate rewards and the other specialized to value delayed rewards [10]. The other model assumes instead a single valuation system that values both immediate and delayed rewards and chooses the option with the higher subjective value [11]. Although the debate is still open, these neural accounts strongly support the idea that idiosyncratic functions of choice behavior are explicitly represented in the brain during task execution.

Given the centrality of intertemporal choice in human decision-making and its alterations in particular clinical populations, however, one notable question is whether it is possible to identify specific neural markers able to reliably predict discounting behavior independently of task execution. A promising method to address this challenge is represented by resting-state functional connectivity MRI (fcMRI). Accordingly, a recent work has provided clear evidence that fcMRI between several cortical networks predicts discounting behavior across subjects [12]. In this study, however, the temporal proximity between the resting-state MRI data collection and the execution of the intertemporal choice task has precluded the possibility to determine whether the relationship between fcMRI and discounting behavior is maintained over time, which is critical for application of these findings to the clinical domain. The motivation for this question appears even more valid when considering the growing body of evidence showing a high test-retest reliability of the fcMRI patterns over months or years, which clearly indicates that fcMRI networks are essentially stable across long periods of time [13–19]. Furthermore, as fcMRI, subjective preferences in TD have been shown to be largely stable over time [1] and to possess the typical properties of personality traits [2,20–22]. Therefore, the fcMRI of the neural circuits involved in TD could represent a reliable long term neural marker of intertemporal choice.

In addition to the time-scale by which fcMRI correlates with discounting behavior, another important question regards the relationship between discounting and impulsivity. The question arises from the observation of a discrepancy between the inconsistent results of the works [23–26] that have directly tested this relationship and the evidence that impulsivity is traditionally considered one of the personality characteristics that contributes the most to a steep discounting function. Indeed, high discounting rate is frequently assumed to be a direct expression or even a direct measure of impulsivity [27,28].

The main aim of the present study is to investigate the intrinsic neural representation of individual differences in TD. By using an intertemporal choice task collected several months after the acquisition of resting-state MRI scans, here we test for the first time whether fcMRI can be used as a long term correlate of individual preferences in economic choice behavior. Notably, in the current study we do not specifically investigate longitudinal changes in discounting behavior, but whether the relationship between rs-fMRI and discounting rate (as measured just once about one year apart) is maintained over time, thus representing a reliable long-term neural marker of economic preferences. In addition, as we study resting-state MRI between and within regions previously associated with the single vs. dual account of temporal discounting, we further examine whether the connectivity-discounting relationship specifically supports one of the two neural model of discounting behavior.

Finally, we address the question of the relationship between discounting rate and individual state/trait differences by conducting a comprehensive non-clinical assessment of personality traits, impulsivity and decision style and by measuring the association between these self-reported measures and independent measures of temporal discounting.

## Materials and Methods

### Ethics Statement

Participants provided written informed consent before the beginning of the experiment, which was approved by the Ethics Committee of the ‘‘G. d’Annunzio’’ University, Chieti (Comitato Etico per la Ricerca Biomedica delle Province di Chieti e di Pescara e dell’Università degli Studi "G. D’Annunzio" di Chieti e Pescara) and was conducted in accordance with the ethical standards of the 1964 Declaration of Helsinki.

### Subjects

Twenty-five right-handed healthy subjects (16 females, mean age = 25.8 years, s.d. = 2.53, range 21–30) participated in the experiment. Each subject completed an fMRI session of eye open resting-state, a behavioral session of an intertemporal choice task and a non-clinical assessment of personality, impulsivity and decision-making style. At the time of the MRI acquisition, participants have never had any previous experience with the intertemporal choice task. The non-clinical assessment and the behavioral session were collected several months (range: 4–18 months, mean: ∼11) after the resting-state MRI acquisition.

### fMRI data collection

During the resting-state scans, subjects lay in the scanner with no experimenter-imposed task other than maintaining central fixation on a cross-hair. Each subject completed 3 resting-state runs for a total of 15 m. Functional T2*-weighted images were collected on a Philips Achieva 3T scanner using a gradient-echo EPI sequence to measure the BOLD contrast over the whole brain (TR = 1869 msec, TE = 25 msec, 39 slices acquired in ascending interleaved order, voxel size = 3.59x3.59x3.59 mm, 64x64 matrix, flip angle = 80°). Structural images were collected using a sagittal M-PRAGE T1-weighted sequence (TR = 8.14 msec, TE = 3.7 msec, flip angle = 8°, voxel size = 1x1x1 mm).

### Intertemporal Choice Task

On each trial participants were asked to choose between two different amounts of money, one immediately available, and one available at different time delays. The immediate choice involved a constant amount of 10€ while the delayed choices were parametrically manipulated across seven different amounts (15€, 25€, 30€, 40€, 45€, 55€ and 60€) and six delay periods (7, 15, 30, 60, 90 and 180 days), for a total of 42 possible choice contingencies. A total of 420 trials, including 10 repetitions per condition, were pseudorandomly distributed across three experimental blocks. Visual presentation of the choice alternatives and recordings of the subjects’ responses were performed using the Mouse Tracker software [29] which also allowed the recording of the kinematics of the mouse movements associated with the choice. Choices options were presented on a 17’’ LCD computer monitor (1285 x 1039 pixels) with a 60 hertz refresh rate at a viewing distance of ∼60 cm.

**Fig 1:**
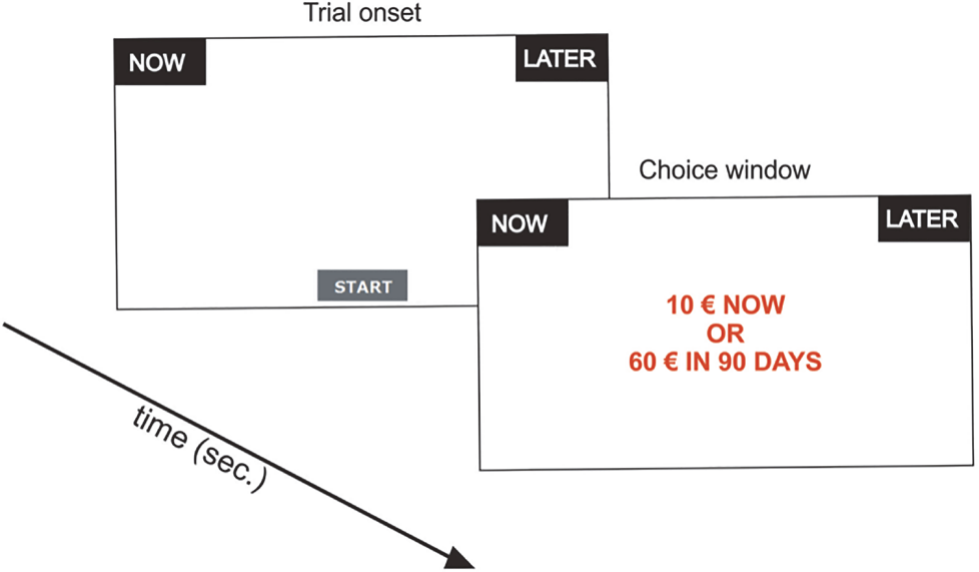
Behavioral paradigm. On each trial, the subjects were instructed to press the “start” button positioned at the central bottom of the screen to visualize the choice options and to express their preference by clicking on the corresponding button (“now” versus “later”).

At the beginning of the first block, an instruction screen was centrally presented to inform subjects on the experimental task and was followed by five training trials. The immediate (“now”) and delayed (“later”) options were presented as written text on a black box (297 x 140 pixels) at the top left and top right of the computer screen, respectively, and remained on the screen for the entire duration of the block. On each trial subjects were instructed to press the “start” button (129 x 72 pixels) positioned at the central bottom of the screen to visualize the choice options and to indicate their preference by clicking on the corresponding button (Fig. 1). Because the immediate amount of reward always corresponded to 10€, only the factorial combination of amount and delay of the delayed option was manipulated across trials.

Participants were explicitly instructed to respond as fast as possible and a pop-out window advised them if their response times were excessively long (>2000 ms). At the beginning of the experiment participants were told that at the end of the session a trial would be randomly selected from their response files and that they would receive a corresponding payment both in terms of amount and time delay. At the end of the experiment the subjects assisted to trial extraction and together with the experimenter consigned an envelope with the corresponding amount of money and indication of the date (in case of delayed payment) and mailing address to the university accounting office.

### Psychophysical analysis of intertemporal choice task

Subject-specific discount rates (k) were estimated using a standard routine also employed in previous works (see [11,12,30] for reference). Specifically, we first estimated the fraction of times the subject chose the future amount over the immediate amount as a function of the objective value of the delayed reward. We then fit the data to a logistic function implemented in Matlab [31] to calculate, for each time delay and subject, a point of subjective equivalence (pse), defined as the amount of money, at that interval, for which that subject would be predicted to choose the smaller-sooner and larger-later rewards with equal frequency. We subsequently estimated the subjective value (SV) for each time delay using the following equation:

$$SV=\frac{10}{pse}$$

where 10 was the amount of money immediately available. This procedure ensured that subjective values were normalized to the immediate amount of money. The subject-specific k parameter was finally estimated by fitting the points of subjective value obtained for each time delay (D) to the well-known hyperbolic function [32–34], defined by the following equation:

$$SV=\frac{1}{\left(1+kD\right)}$$

which provides a widely accepted model to describe the decline in subjective value with increasing time delays.

### Assessment of Personality, Impulsivity, and Decision-Making Style

Personality traits were evaluated using the short Italian version of the Big Five Questionnaire (BFQ-2R) [35]. The BFQ-2R assesses five personality constructs defined by the Five Factor Theory of Personality [36]: extraversion (E), indicating a confident and enthusiastic approach to interpersonal relations; agreeableness (A) or friendliness, the orientation towards altruism and taking care of the other; conscientiousness (C), the tendency to be precise, accurate and persevering; emotional stability or neuroticism (ES), the orientation to control of emotional states and impulses; and intellect or openness to experience (OE), the openness to new ideas, to the values of others and their feelings.

Impulsivity was assessed through the Italian version of the Barratt Impulsiveness Scale (BIS-11) [37,38]. The BIS-11 is a gold-standard questionnaire for the assessment of impulsivity that evaluates three specific dimensions: attentional impulsiveness (AI) involving task-focus, intrusive thoughts, and racing thoughts; motor impulsiveness (MI), involving the tendency to act on the spur of the moment and consistency of lifestyle; and non-planning impulsiveness (N-PI), involving careful thinking and planning and enjoyment of challenging mental tasks.

Finally, decision style was assessed through the Italian version of the General Decision-Making Style Inventory (GDMS) [39]. The GDMS evaluates five different decision trend: rational style (R), characterized by an accurate and logical evaluation of the alternatives; an intuitive style (I) characterized by a dominant influence of impressions and feelings during decision-making; a dependent style (D), characterized by reliance on judgments of others; an avoidant style (A) characterized by attempts to avoid decisions; and finally, a spontaneous style (S), characterized by a sense of immediacy and a tendency to make a decision as soon as possible.

All questionnaires were administered in electronic version through implementation in SuperLab 4.0.7b [40,41] and the association between the individual scores on these tests and discounting rate (k) was assessed through Pearson’s correlations.

### Generic fMRI Pre-processing

All the fMRI analyses were conducted using in-house software developed at the Washington University in Saint Louis. Differences in the acquisition time of each slice in a MR frame were compensated by sinc interpolation so that all slices were aligned to the start of the frame. Functional data were realigned within and across runs to correct for head movement using six-parameters rigid body realignment. To reduce the effects of motion-related confounds on fcMRI measures we used a standard preprocessing technique for the motion correction only on subjects characterized by similar levels of movements, after removing those with larger movements (three subjects). Specifically, image realignment was done by first realigning each frame with the first frame of the BOLD run and then realigning each BOLD run with the average of the first frames of each BOLD run. A whole brain normalization factor was uniformly applied to all frames within a run in order to equate signal intensity across runs. Images were resampled into 3 mm isotropic voxels and warped into 711-2C space, a standardized atlas space [42,43].

### FcMRI pre-processing

FcMRI analyses were computed using previously published methods based on the temporal correlation of the BOLD signal time course recorded at rest [44–46]. After standard pre-processing, several additional preprocessing steps were conducted to remove sources of spurious correlations. First, images were spatially smoothed using a 6-mm FWHM Gaussian filter and temporally filtered using a low-pass filter with a cut-off frequency of 0.1 Hz. Nuisance regressors, including six parameters obtained by rigid body head motion correction (3 translations, 3 rotations), the signal averaged over the whole-brain, the signal averaged over the lateral ventricles, and the signal averaged over a region centered in the deep cerebral white matter, were then removed from the signal using multiple regression. Temporal derivatives of these regressors were also included in the linear model, accounting for time-shifted versions of spurious variance.

### FcMRI analysis

**Fig 2:**
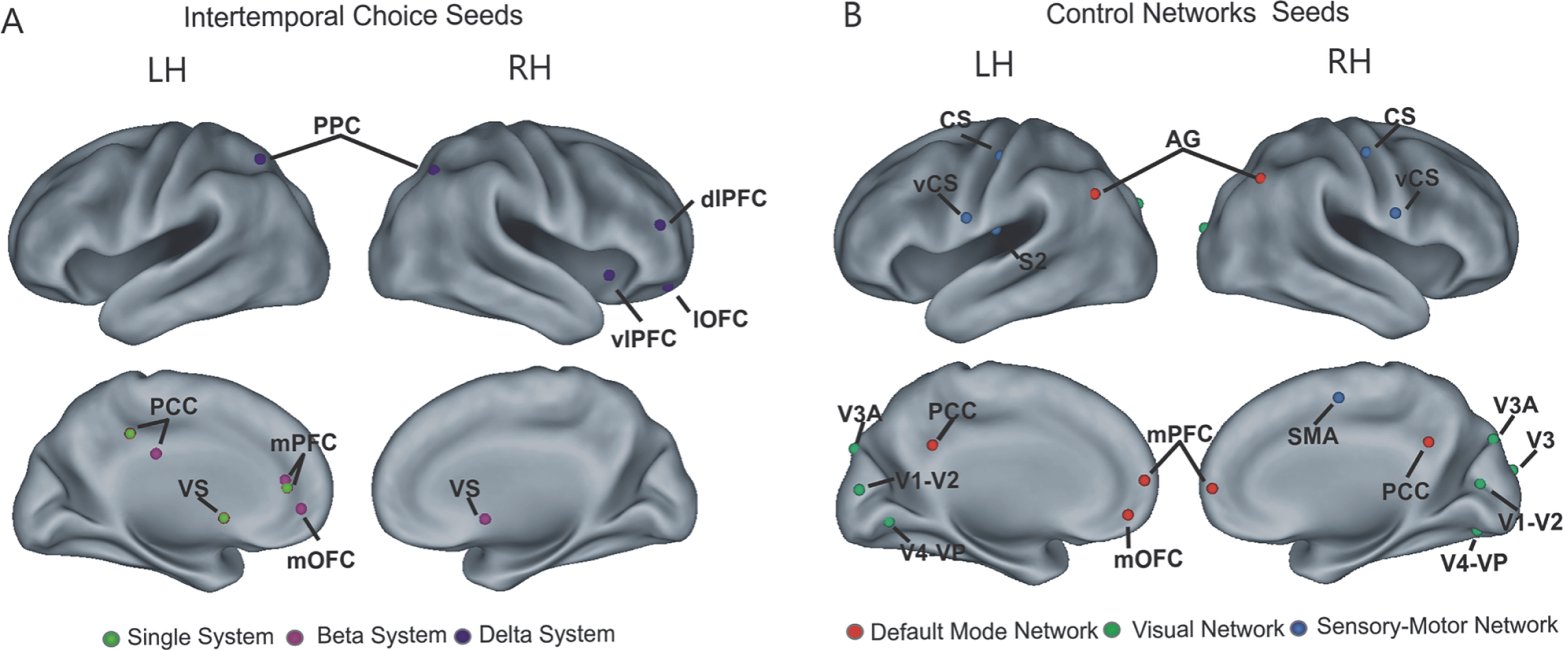
Seed regions used for functional connectivity regional analysis. (A) Seed regions associated with intertemporal choice identified in previous works based on task evoked activity: the single valuation system (green) by Kable and Glimcher (2007) [11], and the β (violet) and δ (blue) systems by McClure et al. (2004) [10]. (B) Control networks seeds: Default Mode Network (DMN, red), Visual Network (VIS, green), and Sensory-Motor Network (SMN, blue) by Hacker et al., (2013) [54]. PCC, posterior cingulate cortex; mPFC, medial prefrontal cortex; VS, ventral striatum, mOFC, medial orbitofrontal cortex; PPC, posterior parietal cortex; dlPFC, dorsolateral prefrontal cortex; lOFC, lateral orbitofrontal cortex; vlPFC, ventrolateral prefrontal cortex; SMA, supplementary motor area; AG, angular gyrus; CS, central sulcus; vCS, ventral central sulcus (seed regions coordinates are reported in Table 1).

The main connectivity analysis was conducted using a classic seed-based approach in which the BOLD time course was first extracted from a set of seed regions identified in previous fMRI studies and then a correlation coefficient was computed between two seeds time courses (regional analysis) or between the time course of a seed and those of all other brain voxels (whole brain analysis). The analysis of the relationship between connectivity and discounting behavior was conducted using linear regressions and correlation analyses. In particular, to investigate whether the relationship between resting-state functional connectivity and discounting behavior specifically supported the single or dual neural valuation accounts for intertemporal choice, we tested whether functional connectivity within and between critical nodes of these systems, as identified in previous fMRI studies, significantly accounted for individual differences in discounting rate. To this aim, we first placed a set of 6 mm radius spheres centered at Talairach (or transformed to Talairach) peak coordinates of regions associated with intertemporal choice in the study by Kable and Glimcher [11], for the single evaluation system, and by McClure and colleagues [10], for the dual valuation system. As shown in Fig. 2*A*, seed regions of the single evaluation system included the posterior cingulate cortex (PCC), the medial prefrontal cortex (mPFC) and the ventral striatum (VS), while seed regions of the β system of the dual valuation account included the same regions of the single evaluation system (mPFC, VS, PCC) with the addition of the medial orbitofrontal cortex (mOFC). Seed regions of the δ system included the posterior parietal cortex (PPC) bilaterally, the right dorsolateral prefrontal cortex (dlPFC), the right lateral orbitofrontal cortex (lOFC), and the right ventrolateral prefrontal cortex (vlPFC) (see Table 1 for seed regions coordinates).

**Table 1:** Seed Regions Coordinates.

Systems	Regions	X	Y	Z
**Single**	PCC	−43	37	12
	mPFC	−3	38	13
	VS	−12	5	1
**β**	PCC	−8	−30	31
	mPFC	−1	38	16
	VS	6	4	−1
	mOFC	−8	42	1
**δ**	rPPC	37	−62	42
	lPPC	−32	−61	42
	dlPFC	41	38	21
	lOFC	23	44	−6
	vlPFC	38	15	−3
**DMN**	rPCC	8	−51	29
	lPCC	−8	−51	29
	rmPFC	2	3	55
	lmPFC	−14	53	13
	mOFC	−1	44	−2
	rAG	45	−66	40
	lAG	−43	−63	32
**SMN**	SMA	1	−10	49
	rVCS	56	−2	23
	lvCS	−57	−8	21
	rCS	37	−18	48
	lCS	−32	−25	46
	S2	−51	−19	20
**VN**	rV3A	11	−85	32
	lV3A	−14	−93	30
	rV4-VP	23	−74	−10
	lV4-VP	−11	−74	−6
	rV1-V2	6	−77	11
	lV1V2	−2	−91	8
	V3	20	−92	18

Following seeds definition, the BOLD signal time series was extracted and averaged across all voxels in each seed region and then a correlation coefficient was estimated from all possible seeds pairs. Correlation coefficients were then Fisher z-transformed and averaged across all possible seeds pairs within (within-network connectivity) and between (between-network connectivity) each network, resulting in three within-network and three between-network coefficients [47]. We next examined the relationship between these correlation coefficients and discounting rate by using the Matlab robust fit function [48,49] to test both linear and robust regressions in which the k parameter was treated as a dependent variable and the functional connectivity coefficient as regressor.

Following linear and robust regressions, we conducted an additional analysis to isolate the specific contributions of each region within a network. In particular, based on the results of the regression analyses, we examined whether all regions within a network equally contributed to the correlation with discounting behavior by using a leave-one-region-out procedure in which each region was sequentially removed from the computation of both within and between-network correlation coefficients and a Pearson’s correlation coefficient was re-estimated each time. In this reiterative procedure, a certain region was considered important for the fc-behavior correlation if its elimination from the computation of the fc-behavior correlation yielded to a non-significant correlation. Notably, because the number of within- and between-network correlations were specific for the number of regions included in the networks, Pearson’s correlations were corrected for multiple comparison using false discovery rate (FDR) [50].

We then examined whether the regions identified through the leave-one-region-out procedure formed a consistent network of functional connectivity by conducting an analysis of the overlap between the whole-brain connectivity maps associated with each of these regions. In particular, we extracted the time course from each of the regions that survived the procedure and computed a voxelwise correlation analysis. Each of the whole-brain connectivity map was then Fisher z-transformed and corrected for multiple comparisons using joint z-score/cluster size thresholds [51] corresponding to z = 3.5 and a cluster size of 11 face-contiguous voxels (p < 0.01). The resulting maps were finally transformed into binary masks including positive values and summed to obtain a single map of connectivity overlap [46,52,53].

We also examined the topographical specificity of the results by testing the association between discounting rate and connectivity measures in a set of control networks, including the default network (DMN), the sensori-motor network (SMN) and the visual network (VIS) (Fig. 2*B*) (see Table 1 for seed regions coordinates), that are not associated with discounting behavior [54]. Seven 6 mm radius regions were drawn at specific Talairach peak coordinates and then a correlation coefficient was estimated for all possible seeds pairs within each network. Correlations coefficients were then Fisher z-transformed, averaged across all possible seeds pairs and finally used as regressors in linear regression analyses in which the discounting rate was the dependent variable.

As a final control analysis, we tested whether the results obtained using *a priori* selected seeds/networks associated with discounting behavior in previous task-evoked fMRI studies could be replicated in a data-driven analysis in which the k parameter was correlated with a whole brain connectivity map estimated from the correlation of each brain voxel with all the others. To this aim, the original grid of 1 voxel size (voxel size = 3.59x3.59x3.59 mm) was first down-sampled by averaging the data of 8 (2x2x2) neighboring voxels and then a consistency map was generated in which each grid element represented the number of connections relevant to behavior. The map was obtained in two steps: first, a Fisher z-transformed Pearson correlation coefficient was used to estimate in each subject a cross-correlation matrix of connectivity between each grid element and all the others. Second, an across-subject Pearson’s correlation was computed between the significant functional connectivity elements from step 1 (one sample t-test against 0, p = 0.05) and the k parameter. A consistency map was finally generated in which each element represented the number of significant connections with all the other elements whose functional connectivity significantly correlated with behavior (p = 0.05, Bonferroni corrected). In order to graphically highlight the seeds with the maximum number of connections correlated with the k parameter (“hubs of behavior”), a value corresponding to the mean plus three standard deviations (approximately 99% of the data) was used to threshold the map.

## Results

### Intertemporal choice task

**Fig 3:**
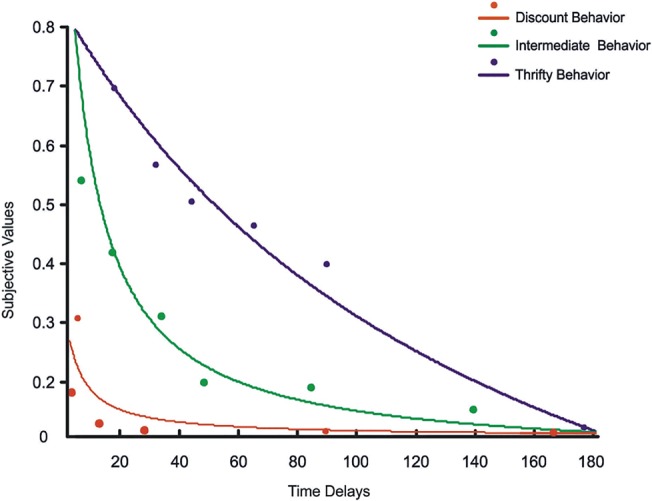
Discounting functions from three representative subjects obtained by fitting the subjective values for each time delay with the hyperbolic function: SV = 1/(1+k D).

As expected, the k parameter reflecting discounting rate showed a large inter-subject variability, with estimates that varied from a minimum of 0.036 to a maximum of 1.56 (mean = 0.211; standard deviation = 0.329) (see Fig. 3 for three representative subjects).

In addition to individual estimates of the k parameter, we controlled that our intertemporal choice paradigm was effective to elicit the classic trade-off between reward amount and delay by conducting a repeated measures ANOVA with reward amount (15€, 25€, 30€, 40€, 45€, 55€ and 60€) and time delay (7, 15, 30, 60, 90 and 180 days) as factors on both execution times of mouse trajectories and choice distribution. Consistent with previous findings, we observed a main effect of time delay (F_5, 120_ = 7.129, p < 0.01), with longer execution times for increasingly longer time delay, a main effect of reward amount (F_6, 144_ = 4.598, p < 0.01), with faster execution times for increasingly larger reward amount and a significant interaction of time delay by reward amount (F_30, 720_ = 3.399, p < 0.01). The interaction was explained by particularly short execution times for conditions in which either the reward amount was large or the time delay was short, i.e. easy decisions, compared to conditions in which both the reward amount and time delay were intermediate, i.e. difficult decisions.

Consistent with the results on execution times, the analysis of choice frequency indicated that the percentage of delayed choices progressively increased as the reward amount increased and the time delay decreased. Moreover, a robust increase of the delayed choices was observed for conditions of intermediate time delay and reward amount compared to conditions of small reward amount or long time delay. This was statistically confirmed by a main effect of time delay (F_5, 120_ = 38. 078, p < 0.01) and reward amount (F_6, 144_ = 72.193, p < 0.01) and by a significant interaction between them (F_30, 720_ = 5.250, p < 0.01) on the percentage of delayed choices. These results were important to confirm that subjects’ behavior was significantly modulated by both the reward amounts and the reward delays used in the current paradigm as well as by their interaction.

### Relationship between discount rate and fcMRI

To investigate whether discounting behavior could be predicted by intrinsic functional connectivity of those brain circuits that have been previously associated with intertemporal choice behavior, we conducted a series of simple and robust regressions in which discount rate was treated as dependent variable and functional connectivity as regressor.

**Fig 4:**
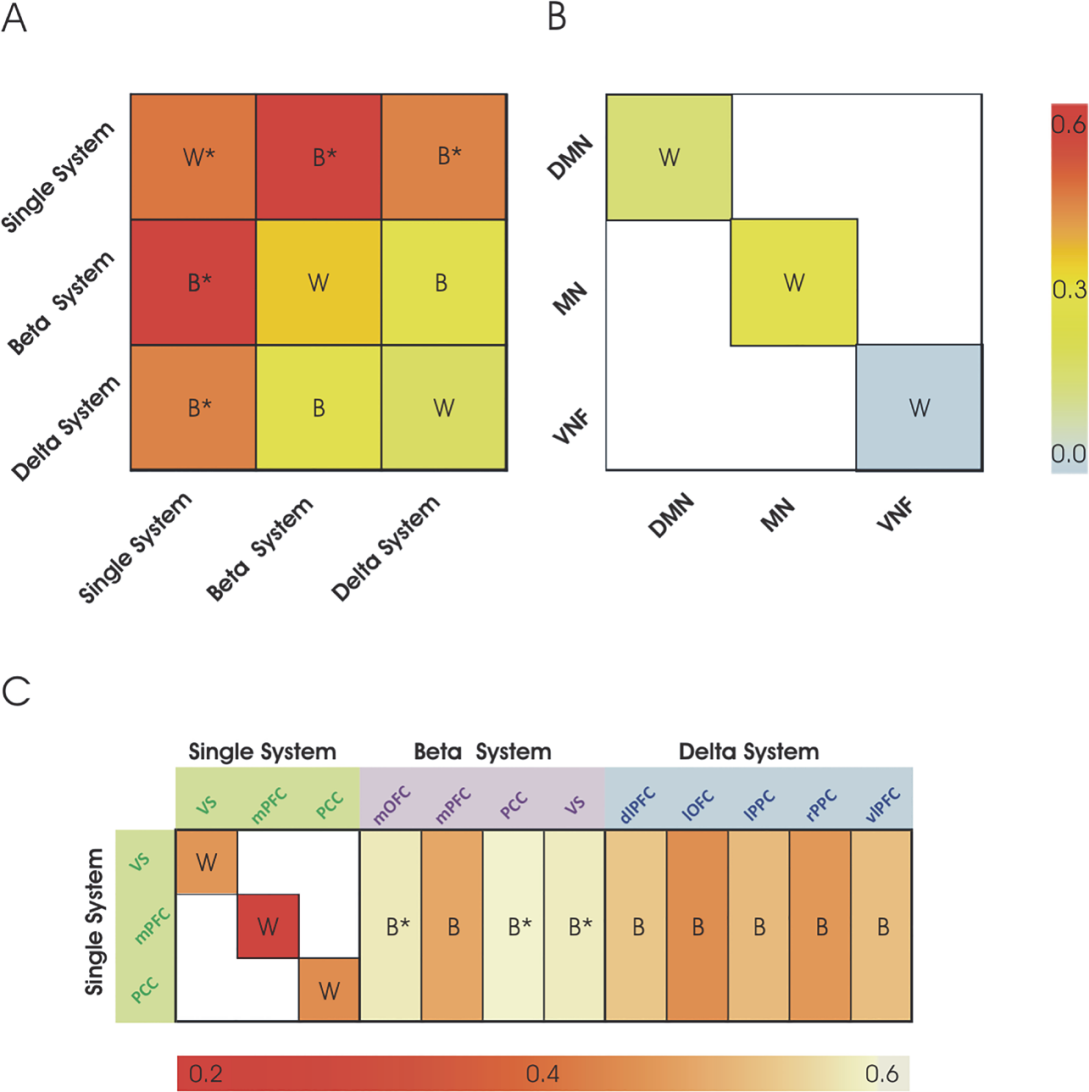
FcMRI-behavior correlation matrices. (A) Relationship between discounting rate and connectivity within- (W) and between- (B) the Single, the Beta and the Delta systems. Red/orange colors indicate positive correlation (within single system; between Single system and both β and δ systems), yellow color indicates non-significant correlations (within and between β and δ systems). (B) Relationship between discounting rate and connectivity within control networks (Default Mode Network, DMN; Sensory-Motor Network, SMN; Visual Network, VIS). Yellow/pale blue colors indicate absence of statistically significant correlations. (C) Leave-one-region-out correlation matrix. Red/orange colors indicate a significant weight of the region in the fcMRI-behavior correlation as expressed by a significant drop in the correlation (0.2–0.3 Pearson’s correlations values) within the Single system (all regions) and between the Single system and both the Beta (mPFC) and the Delta system (all regions). Yellow/white colors indicate a low weight of the region in maintaining the fcMRI-behavior correlation as expressed by a value of the fcMRI-behavior correlation that remained high (0.4–0.6 Pearson’s correlations) when excluding the current region from the computation of the fcMRI–behavior correlation. Regional labels as in Fig. 2.

Regression analyses on within-network connectivity showed that discounting behavior was significantly correlated with the internal connectivity of the single valuation system (beta = 0.43, p = 0.04; effect size = 0.59), whereas it was marginally correlated (beta = 0.40, p = 0.051) or not correlated (beta = 0.20, p = 0.18) with the internal connectivity of the β and δ dual valuation systems, respectively (see Fig. 4*A*). Notably, only the within-network connectivity of the single evaluation system survived the robust regression analysis (Single system: beta = 0.26, p = 0.04; β system: beta = 0.26, p = 0.07; δ system: beta = 0.17, p = 0.14). Therefore, although the single and the β system are defined by partially overlapping anatomical regions and their spontaneous BOLD activity shows strong temporal correlation (mean value = 0.39 Pearson’s correlation), only the within single-valuation system significantly correlated with discounting behavior. Moreover, the between-network connectivity of the single system with both the β and δ systems significantly correlated with discounting behavior (Single-β, linear regression: beta = 0.58, p = 0.003, effect size = 0.89; robust regression: beta = 0.28, p = 0.004; Single-δ, linear regression: beta = 0.43, p = 0.03, effect size = 0.58; robust regression: beta = 0.20, p = 0.041) (Fig. 4*A*). Note that the between-network correlation of the single and the β systems was somehow expected based on their partial anatomical overlap (with the exception of mOFC). All the regression analyses indicated a positive relationship between discounting rate and functional connectivity, so that stronger connectivity was associated with higher discounting behavior.

To control the spatial specificity of these findings we selected a set of well-defined cortical networks that are not typically associated with intertemporal choice behavior, including the sensori-motor (SMN), the visual (VIS) and the default mode (DMN) network (see Fig. 2*B*) and tested whether their internal correlation significantly correlated with discounting behavior. The results of linear regressions (Fig. 4*B*) showed no significant association between functional connectivity within these control networks and the k parameter (DMN: beta = 0.13, p = 0.54; SMN: beta = −0.11, p = 0.58; VIS: beta = −0.22, p = 0.30), indicating that a significant relationship was only observed with systems that have been specifically associated with discounting behavior in task-evoked fMRI studies [10,11]. This was statistically confirmed by a direct comparison between the fc-behavior correlation coefficients of the relevant and control networks using the Lisrel 9.10 Student Edition software [55]. With the exception of the non-significant difference between the DMN and the single system (*X* ^2^ = 2.54 p = 0.1) and the DMN and the δ-Single between-network connectivity (X^2^ = 1.53 p = 0.2) which was likely explained by the relatively large overlap between the DMN and the single system, the results showed that the difference between the fc-behavior correlation coefficients of the two sets of networks was significant (or marginally significant) (Single-SMN: *X* ^2^ = 3.6, p< 0.05; Single-VN: *X* ^2^ = 5.69, p< 0.05; β-Single –DMN: *X* ^2^ = 4.78 p< 0.05; β-Single –SMN: *X* ^2^ = 6.64 p< 0.01; β-Single –VN: X^2^ = 9.56 p< 0.01; δ-Single – SMN:: *X* ^2^ = 3.38 p = 0.06; δ-Single – SMN:: *X* ^2^ = 5.52 p< 0.05).

Following linear regressions, to identify the brain regions of the single and dual valuation systems that maximally contributed to the correlation with discounting behavior, we carried out a leave-one-region-out procedure combined to correlation analyses (Fig. 4*C*) by evaluating the drop in statistically significant correlations. The results indicated that all the three regions of the single valuation system (mPFC, PCC and VS) significantly contributed to the correlation between the internal coupling of this system and behavior. Concerning the dual valuation systems, while only the removal of the mPFC of the β system led to a non-significant Single-β between-network fc-behavior correlation, all regions of the δ system (lOFC, right and left PPC, vlPFC, and dlPFC) were critical for the fc-behavior correlation, i.e. their elimination yielded to a non-significant Single-δ between-network fc-behavior correlation. Importantly, all the regions whose removal led to a non-significant correlation had an associated p-value >0.1, while significant correlations were always associated with a p-value < 0.01. It is worth noting that the mOFC, which is the only region that is not shared by the β and the single system, did not represent a key region for the Single-β relationship with behavior (Fig. 4*C*).

**Fig 5:**
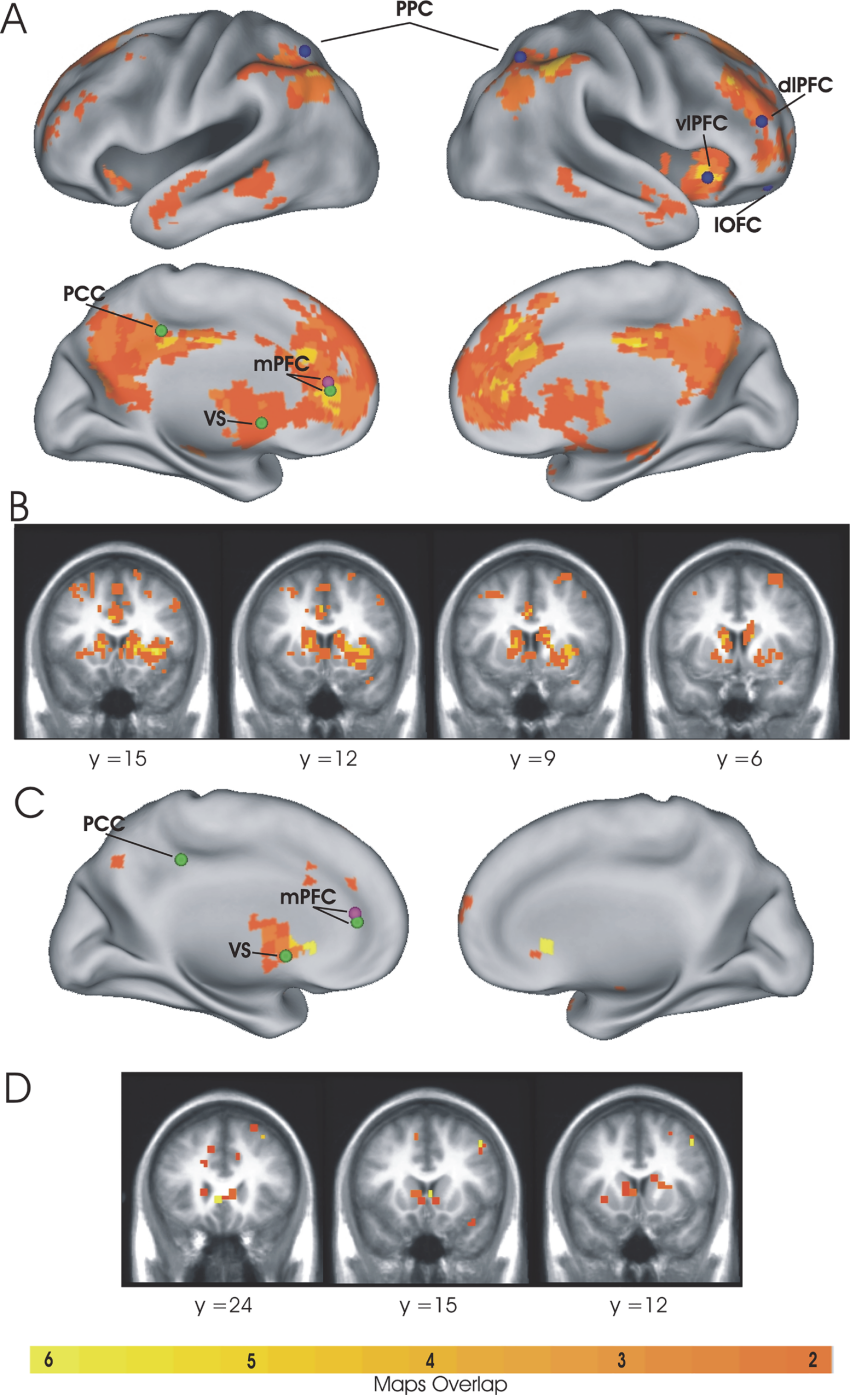
Whole-brain connectivity analysis. Overlap between the whole-brain fcMRI maps associated with each of the nine seed regions identified in the leave-one-region-out analysis is superimposed over an inflated view of the brain surface (A) and on coronal slices of the brain volume (B). The map shows consistent overlap (yellow color represents the higher value of overlap) between the different whole brain fcMRI maps, thus indicating that the 9 regions form a resting-state functional connectivity network for discounting behavior. (C) The whole-brain map of the fcMRI-behavior correlation superimposed over an inflated view of the brain surface (C) and on coronal slices of the brain volume (D) confirmed the crucial role of the ventral striatum and medial prefrontal cortex in predicting discount rate. Seeds labels and colors as in Fig. 2.

Based on the idea that these regions constitute a core resting-state functional connectivity network for discounting behavior, we conducted an additional correlation analysis of fcMRI in which these regions were considered as a part of a single neural system. A functional connectivity index of the internal correlation within these regions was first estimated and then a regression analysis was used to test the relationship with discounting behavior. Results showed that both linear and robust regressions were highly significant (linear regression: beta = 0.60, p = 0.001, effect size = 0.94; robust regression: beta = 0.20, p = 0.004) thus indicating that the internal connectivity of the identified regions significantly correlated with discounting behavior. The presence of a significant relationship with behavior, however, did not indicate that these regions form a resting-state intrinsic network *per se* (i.e. independently of behavior), as significant correlation with behavior could result from very marginal oscillations of the within-network correlation values around 0. Therefore, to assess whether these nine regions form an intrinsic functional connectivity network regardless of behavior, we first computed a whole-brain connectivity map for each of these regions and then examined whether they shared significant connectivity through maps overlap. As shown in Fig. 5*A* and *B*, a consistent overlap was observed between the different whole brain connectivity maps, thus indicating that they form a resting-state functional connectivity network for discounting behavior.

As a last control analysis, we also conducted a data-driven analysis in which the relationship between functional connectivity and discounting behavior was examined over the whole brain with no seed regions as a reference. As shown in Fig. 5*C* and *D*, the results of this analysis confirmed that the most important connectivity hubs associated with discounting behavior corresponded to regions in the ventral striatum and medial prefrontal cortex.

### Relationship between discounting rate and state/trait individual differences

We next conducted a systematic analysis of the relationship between intertemporal choice behavior and other potentially-related behavioral variables by testing the presence of significant correlations between inter-subject variability in the k parameter and personality traits, impulsiveness and decision style, as assessed by the Big Five Questionnaire (BFQ-2R) [35], the Barratt Impulsiveness Scale (BIS-11)[37,38], and the General Decision-Making Style Inventory (GDMS) [39], respectively.

As expected, although in the non-pathological range, a sufficient inter-subjects variability was observed, especially for dimensions of impulsivity and decision style (see Table 2). As indicated in Table 3, however, no significant correlations (Pearson’s) were observed between discounting rate and any of the evaluated dimensions of personality, impulsiveness and decision-making style.

**Table 2:** Mean scores on BFQ-2r, BIS-II, and GDMS.

BIG FIVE QUESTIONNAIRE	
EXTRAVERSION	3.4±0.44
CONSCIENTIOUSNESS	3.9±0.56
NEUROTICISM	4.1±0.41
AGREEBLENESS	3.1±0.71
OPENESS TO EXPERIENCE	4.0±0.33
**BARRATT IMPULSIVENESS SCALE II**	
ATTENTIONAL IMPULSIVITY	14.8±2.93
MOTOR IMPULSIVITY	19.0±3.35
NON-PLANNING IMPULSIVITY	19.0±3.35
TOTAL IMPULSIVITY	56.8±8.51
**GENERAL DECISION MAKING STYLE TEST**	
RATIONAL STYLE	16.4±2.69
INTUITIVE STYLE	16.6±3.11
DEPENDANT STYLE	13.1±3.41
AVOIDANT STYLE	12.4±3.23
SPONTANEOUS STYLE	15.8±2.85

**Table 3:** Correlations between BFQ-2r, BIS-11, GDMS individual scores and both discount rate parameter (k) and functional connectivity coefficients.

	Discount	Single	β	δ	Single x β	Single x δ	Β x δ
	Rate (k)	System	System	System	Systems	System	Systems
**EXTRAVERSION (BFQ-2r)**	r = 0.14	r = 0.10	r = −0.16	r = 0.10	r = 0.10	r = 0.15	r = 0.05
	p = 0.48	p = 0.602	p = 0.43	p = 0.602	p = 0.618	p = 0.474	p = 0.79
**CONSCIENTIOUSNESS (BFQ-2r)**	r = 0.06	r = −0.26	r = −0.03	r = −0.10	r = −0.04	r = 0.17	r = 0.03
	p = 0.75	p = 0.20	p = 0.91	p = 0.65	p = 0.85	p = 0.41	p = 0.89
**NEUROTICISM (BFQ-2r)**	r = −0.125	r = −0.29	r = −0.39	r = 0.04	r = −0.11	r = −0.06	r = −0.29
	p = 0.552	p = 0.17	p = 0.06	p = 0.84	p = 0.59	p = 0.78	p = 0.16
**AGREEBLENESS (BFQ-2r)**	r = 0.27	r = 0.06	r = 0.13	r = −0.09	r = 0.33	r = 0.34	r = 0.35
	p = 0.18	p = 0.78	p = 0.55	p = 0.66	p = 0.11	p = 0.09	p = 0.09
**OPENESS TO EXPERIENCE (BFQ-2r)**	r = 0.54	r = 0.24	r = 0.33	r = −0.03	r = 0.38	r = 0.14	r = 0.13
	p = 0.01	p = 0.25	p = 0.11	p = 0.90	p = 0.06	p = 0.50	p = 0.54
**ATTENTIONAL IMPULSIVITY (BIS-11)**	r = 0.23	r = 0.28	r = 0.40	r = 0.07	r = 0.34	r = 0.03	r = −0.10
	p = 0.25	p = 0.18	p = 0.05	p = 0.76	p = 0.10	p = 0.90	p = 0.62
**MOTOR IMPULSIVITY (BIS-11)**	r = 0.21	r = 0.36	r = 0.36	r = −0.02	r = 0.34	r = 0.16	r = 0.16
	p = 0.31	p = 0.08	p = 0.08	p = 0.91	p = 0.10	p = 0.45	p = 0.45
**NON-PLANNING IMPULSIVITY (BIS-11)**	r = −0.18	r = 0.09	r = 0.09	r = −0.06	r = −0.10	r = −0.11	r = 0.08
	p = 0.38	p = 0.67	p = 0.68	p = 0.79	p = 0.63	p = 0.59	p = 0.70
**TOTAL IMPULSIVITY (BIS-11)**	r = 0.07	r = 0.28	r = 0.32	r = −0.02	r = 0.20	r = 0.01	r = 0.07
	p = 0.73	p = 0.17	p = 0.12	p = 0.94	p = 0.34	p = 0.95	p = 0.75
**RATIONAL STYLE (GDMS)**	r = 0.17	r = 0.23	r = 0.40	r = −0.02	r = 0.40	r = 0.25	r = 0.24
	p = 0.39	p = 0.27	p = 0.05	p = 0.93	p = 0.05	p = 0.22	p = 0.25
**INTUITIVE STYLE (GDMS)**	r = 0.18	r = 0.23	r = 0.29	r = 0.15	r = 0.33	r = 0.24	r = 0.27
	p = 0.37	p = 0.27	p = 0.15	p = 0.48	p = 0.10	p = 0.24	p = 0.20
**DEPENDANT STYLE (GDMS)**	r = 0.01	r = −0.02	r = −0.01	r = −0.02	r = −0.10	r = 0.06	r = −0.16
	p = 0.96	p = 0.93	p = 0.96	p = 0.94	p = 0.63	p = 0.79	p = 0.45
**AVOIDANT STYLE (GDMS)**	r = −0.05	r = 0.02	r = 0.03	r = 0.01	r = −0.01	r = −0.03	r = −0.13
	p = 0.80	p = 0.93	p = 0.89	p = 0.96	p = 0.95	p = 0.90	p = 0.53
**SPONTANEOUS STYLE (GDMS)**	r = 0.01	r = −0.18	r = 0.04	r = 0.01	r = −0.03	r = −0.06	r = −0.12
	p = 0.94	p = 0.39	p = 0.85	p = 0.95	p = 0.90	p = 0.79	p = 0.56

In addition to correlations between different behavioral variables, we also examined whether functional connectivity networks associated with discounting behavior exhibited significant correlations with non-clinical dimensions of personality, impulsiveness and decision-making style. As for individual state or trait behavioral differences, the results indicated no significant correlation (Person’s) between inter-subject variability in any of the evaluated dimensions and connectivity within and between intertemporal choice networks (see Table 3), thus confirming the specificity of the relationship between functional connectivity and discounting rate.

These findings were particularly relevant for the question of the relationship between discounting and impulsivity, as they argued against the widely held assumption that high discounting rate is a direct expression of impulsivity.

Notably, we also controlled that the absence of correlation was not explained by poor inter-subject variability in impulsivity scores by dividing our experimental sample into two subgroups based on impulsivity scores and testing subgroup differences. The results indicated a significant difference in the impulsivity score (t = 6.20, p < 0.01), which was not paralleled by a difference in discounting rate (t = −1.25, p = 0.23). Importantly, the reverse pattern was found when dividing subjects based on the k parameter (k parameter subgroups difference: t = 2.18, p < 0.05; impulsivity subgroups difference: t = 0.04, p = 0.96).

Overall, although the absence of correlation is a null result, the finding that individual variability in discounting rate is significantly associated with functional connectivity within and between specific brain networks but not with other individual differences in impulsivity, personality traits or decision-making styles suggests that discounting behavior is a particularly unique behavioral construct that is explicitly represented in the human brain both at the level of task-evoked activity of specific brain regions and at the level of their functional communication at the resting-state.

## Discussion

The present findings indicate that discounting behavior, as measured during an intertemporal choice task, was significantly correlated with resting-state functional connectivity within and between specific neural networks classically associated with discounting behavior.

The relationship between resting-state functional connectivity and discounting behavior has been already evidenced in recent works on both clinical [7] and non-clinical [12] subjects but to the best of our knowledge this is the first study showing that functional connectivity can be a reliably long-term correlate of individual differences in discounting behavior. In our study, in fact, resting-state was measured through fMRI several months before the collection of the behavioral data on the intertemporal choice task. This is remarkable given the multitude of events occurred between the two measures that have possibly introduced uncontrolled variability and thus limited statistical power.

Concerning the neural systems implicated, our results showed that both the single system [11] and the two systems of the dual valuation account [10] play a role in intertemporal choice behavior as both the internal correlation of the single system (within-network) and its coupling with the β and δ systems showed a positive relationship with discounting rate: stronger connectivity (i.e., increased communication between specific nodes of the networks defined through task-evoked activity during intertemporal choice) was associated with steeper discounting function (i.e., decreased willingness to accept delayed rewards). This result is in accord with several lines of evidence. First, the findings of Li and colleagues that discounting rate is positively correlated with functional connectivity within a series of networks defined by task-evoked regressors associated with valuation and choice processes during intertemporal choice. Second, the findings on temporal discounting and functional connectivity in aging. In particular, whereas it is widely accepted that the progressively farsighted behavior in aging is associated with progressively less steeper discounting functions [56–59], independent lines of evidence indicate that aging is associated with a progressive decrease of functional connectivity within specific brain circuits including the default network [60], the motor network [61], and the network involved in emotional evaluation [62]. More relevantly, a recent work on older adults by Han and colleagues reported a positive correlation between discounting rate and rs-fcMRI of the frontal insula with the ventro-medial prefrontal cortex and middle temporal cortex [63]. The results of these studies are in keeping with our findings of decreased resting-state functional connectivity in farsighted subjects. Third, the evidence of dysfunctional hyper-connectivity in pathological populations [52,64,65]. More pertinent to the present context, a recent study on cocaine-dependent individuals [7] found an increase in resting-state connectivity between frontal regions associated with self-regulation (anterior cingulate cortex and dorsolateral prefrontal cortex) which was positively correlated with temporal discounting.

Importantly, the relationship of the single and dual valuations systems with behavior showed a strong topographical specificity, as only their functional connectivity, and not that of a series of control networks, significantly correlated with discounting rate. This indicates that the role of these networks in discounting behavior is not limited to task execution but also involves their functional interaction at rest.

Concerning the debate on the two neural accounts for immediate and delayed rewards, instead, our functional connectivity results are not able to draw definitive conclusions. Indeed, even though the internal coupling of the single system was sufficient to connectivity-behavior relationship (within-network connectivity), also its functional coupling (between-network connectivity) with the β and δ systems of the dual valuation account significantly correlated with discounting rate. Noteworthy, although the β and δ systems are classically conceptualized in an antagonist relationship, the positive values observed in both the between-network regressions indicated no dissociation in their behaviorally-predictive functional coupling with the single system. Furthermore, we speculate that the functional role of the nine regions identified in our study through the leave-one-region-out procedure can be better described using the recent model of temporal discounting proposed by Peters and Büchel [66] that describes TD as constituted by several cognitive components, each predominantly associated with specific brain structures (see also [34] for a related componential model of TD). In this model, TD would be the result of three main cognitive components: one associated with the evaluation of the available decision options, one associated with self-control during the decision process and one associated with the representation of decision outcomes (prospection). The evaluation mechanism involves the computation and representation of the subjective value of the available choice options and is associated with brain structures such the ventral striatum, the ventro-medial prefrontal cortex and the posterior cingulate cortex that have been traditionally implicated in reinforcement learning and reward processing [30,67–73]. The self-control component instead is conceptualized as a modulatory component of the intertemporal choice process that biases behavior toward delayed but larger gratification via direct or indirect top-down influence on the evaluation system. This self-control component is thought to be controlled by regions of the dorsolateral prefrontal cortex (dlPFC) which has been associated with the development of self-control during adolescence [74–76], with modulation of the ventro-medial prefrontal cortex valuation system during deployment of self-control in decision-making [77] and with causal influence of self-control processes in intertemporal choice [78]. Finally, the third component of the model is thought to support future-minded decision-making through mechanisms of episodic future thinking and prospection. This component, which allows to reduce the rate of discounting through anticipation and imagination of delayed consumption, is associated with medial temporal lobe structures such the hippocampus and with lateral parietal regions [66,79–81].

An alternative framework for interpreting our findings posits that the brain networks for intertemporal choice are differentially recruited during difficult vs. easy decisions and depending on uncertainty of the outcomes. In this context, Daw et al. (2005) [82] proposed that during choice two neuronal controllers, one inflexible but rapid (associated with model-free reinforcement learning) and another more flexible but also more cognitively demanding (associated with model-based reinforcement learning), continuously compete for selection based on task uncertainty. A recent extension of this idea is that the (simpler) model-free controller might act by default and recruit the model-based controller only when uncertainty is too high and it is necessary to lower it before the choice [83–85]. According to the latter hypothesis, a core brain network implementing model-free value computations could be recruited for any choice. When the uncertainty is too high, additional brain areas would be recruited that improve the evaluation by implementing model-based computations, which are often associated to "cognitive search" and episodic future thinking. According to this hypothesis, the single and the β systems could be more directly responsible of evaluating the outcomes while the δ system might exert a modulatory role when needed (and there is enough time). The empirical validity of this hypothesis however remains to be tested in the future.

The second major point we addressed in this work concerns the role of impulsivity in steep discounting functions as it has been suggested that the choice of immediate rewards can be guided by an impulsive system triggered by the temporal proximity of the stimuli [27]. The linkage between discounting and impulsiveness, however, has been often treated as an a-priori assumption without any direct testing. In Ainslie (1975) and Monterosso and Ainslie (1999), for example, the authors explicitly claim that the k parameter can serve as an index of impulsive behavior but they did not report any study in which the relationship between trait impulsivity and steep discounting function was explicitly tested independently of intertemporal choice[27,28]. Also in more recent years, the relation between discounting behavior and impulsivity has been widely assumed without any direct testing [12,86,87].

This evidence can be partially reconciled if one considers that early studies on impulsivity used a “delayed gratification” paradigm [88]. For example, in the classic study by Mischel et al (1988) [89], the experimenter showed preschool children a small food reward, offering them the opportunity to obtain a higher food reward if they were able to wait until the experimenter returned to the room without consuming the food. While this task can be considered as formally equivalent to the choice between an immediate and a later reward, only one option is presented immediately and is under the children’s focus of attention. This might more easily trigger impulsive responses, whereas the role of impulsivity might be less relevant in the paradigm we used (and which is more commonly used in fMRI experiments) because both options are presented simultaneously.

Here we explicitly tested the relation between impulsivity and temporal discounting by collecting independent measures of impulsivity (BIS-11) and economic choice behavior (intertemporal choice task), and we found no correlation between these two measures.

Overall, however, the role of trait impulsivity in intertemporal choice is still not well understood, and has often driven to quite inconsistent results. This inconsistence could be due to several possible reasons. First, some of the studies reporting significant correlations between impulsivity and discount rate were conducted in clinical populations of addicted individuals (cocaine, heroin or opioid-dependent individuals) [26,90,91]. These clinical conditions are frequently associated with severe impulsivity, as testified by pathological impulsivity scores in standard tests, and generalizing the results obtained in the clinical domain to the general population might be incorrect. Some other studies have obtained quite low values of correlation (r = ∼ 0.20) despite very large samples of participants (n = ∼ 100), which may result by spurious correlations [91]. In other cases, impulsivity has been evaluated through many different questionnaires, obtaining positive correlations only in a small part of them [23], which speaks against the generality of the findings. Notably, moreover, in self-report measures of impulsivity, people have to recognize and report their behavior, and this could be an inaccurate description of their actual behavior. On the other hand, indirect behavioral paradigms such the intertemporal choice or the stop signal task [25,92] provide objective and very specific measures of certain components of what is commonly defined as impulsivity [25]. As a consequence, self-report and behavioral measures could assess different forms of impulsivity [93].

### Conclusion

In summary, our work provides the first evidence that resting-state functional connectivity can represent a reliable long-term correlate of discounting behavior. Our findings also provide new insights on the neural basis of temporal discounting suggesting the existence of a unique intrinsic system responsible of the representation of the main components of intertemporal choice: evaluation, self-control and episodic prospection. Finally, our study does not support a relationship between self-reported measure of impulsivity and delay discounting.
